# Effects of insulin therapy optimization with sensor augmented pumps on glycemic control and body composition in people with cystic fibrosis-related diabetes

**DOI:** 10.3389/fendo.2023.1228153

**Published:** 2023-08-31

**Authors:** V. Grancini, G. Alicandro, L. L. Porcaro, L. Zazzeron, A. Gramegna, L. C. Morlacchi, V. Rossetti, A. Gaglio, V. Resi, V. Daccò, F. Blasi, E. Orsi

**Affiliations:** ^1^ Diabetes Unit, Fondazione IRCCS Ca’ Granda Ospedale Maggiore Policlinico, Milan, Italy; ^2^ Department of Pathophysiology and Transplantation, University of Milan, Milan, Italy; ^3^ Pediatrics, Gastroenterology, Hepatology, Pediatric Transplantation and Cystic Fibrosis Unit, Fondazione IRCCS Ca’ Granda Ospedale Maggiore Policlinico, Milan, Italy; ^4^ Respiratory Unit and Cystic Fibrosis Adult Center, Fondazione IRCCS Ca’ Granda Ospedale Maggiore Policlinico, Milan, Italy

**Keywords:** cystic fibrosis, cystic fibrosis related diabetes, sensor augmented pumps, insulin therapy optimization, insulin pumps, insulin

## Abstract

**Objective:**

Cystic fibrosis (CF)-related diabetes (CFRD) resulting from partial-to-complete insulin deficiency occurs in 40-50% of adults with CF. In people with CFRD, poor glycemic control leads to a catabolic state that may aggravate CF-induced nutritional impairment and loss of muscle mass. Sensor augmented pump (SAP) therapy may improve glycemic control as compared to multiple daily injection (MDI) therapy.

**Research design and methods:**

This non-randomized clinical trial was aimed at evaluating the effects of insulin therapy optimization with SAP therapy, combined with a structured educational program, on glycemic control and body composition in individuals with insulin-requiring CFRD. Of 46 participants who were offered to switch from MDI to SAP therapy, 20 accepted and 26 continued the MDI therapy. Baseline demographic and clinical characteristics were balanced between groups using a propensity score-based overlap weighting procedure and weighted mixed-effects regression models were used to estimate changes in study outcomes.

**Results:**

After 24 months changes in HbA1c were: -1.1% (-12.1 mmol/mol) (95% CI: -1.5; -0.8) and -0.1% (-1 mmol/mol) (95% CI: -0.5; 0.3) in the SAP and MDI therapy group, respectively, with a between-group difference of -1.0 (-10 mmol/mol) (-1.5; -0.5). SAP therapy was also associated with a decrease in mean glucose (between group difference: -32 mg/dL; 95% CI: -44; -20) and an increase in TIR (between group difference: 19.3%; 95% CI 13.9; 24.7) and in fat-free mass (between group difference: +5.5 Kg, 95% CI: 3.2; 7.8).

**Conclusion:**

Therapy optimization with SAP led to a significant improvement in glycemic control, which was associated with an increase in fat-free mass.

## 1 Introduction

Cystic fibrosis (CF) is a life-threatening genetic disease caused by mutations of the CF transmembrane conductance regulator (CFTR) gene. The gene encodes a protein channel expressed in many organs including the pancreas and the lung. The main clinical manifestations of the disease are in fact pancreatic insufficiency and progressive lung disease. Pancreatic insufficiency is present since early childhood in ~80% of this population and requires high energy-density diet with no restrictions on fat and carbohydrate content and pancreatic enzyme replacement therapy. Lung disease is sustained by recurrent respiratory infections and represents the main cause of death in these individuals. However, the recent availability of highly-effective CFTR modulators is going to radically change the natural history of CF.

CF also affects the endocrine function of the pancreas and patients develop a unique form of diabetes, defined as CF-related diabetes (CFRD), which differs from both type 1 and type 2 diabetes mellitus. It is the most common extra-pulmonary CF complication, with a prevalence that is strictly age-related, raising from ~5% in the pediatric population to ~50% in adults, with a further 35% of people having impaired glucose tolerance (IGT) (1, 2).

Dysregulation of glucose metabolism in CF is caused by a progressive decline in insulin secretion, but β-cell loss due to self-digestion of endocrine tissue by trapped pancreatic enzymes is not the only pathological mechanism (3–5). The CFTR protein has been detected also on β-cell surface and its mutations have been associated to alterations in transmembrane potential, accumulation of altered CFTR in the rough endoplasmic reticulum and increased intracellular oxidative stress, leading to premature β-cell apoptosis and insulin deficiency (3).

Conversely, other studies suggest that CFTR may not be expressed in human pancreatic endocrine cells (6) CFRD has been associated with the typical microvascular complications of diabetes mellitus, albeit with different prevalence from type 1 and type 2 diabetes (7–10): retinopathy and microalbuminuria are diagnosed in about 15% of adults with CFRD, while neuropathy and gastroparesis can be detected in more than 50% of them. In terms of long-term clinical outcomes, CFRD has been related to a clinically significant decline in lung function, a decreased survival from lung disease and to a poorer nutritional status (6, 11–15).

Currently, the only recommended treatment for CFRD is insulin therapy, with an early start being associated with a significant improvement in disease prognosis (2, 12, 14–19).

However, insulin treatment may contribute to the multidrug burden of care for people with CF and, our clinical experience has been that, in many cases, multiple daily injection (MDI) regimens may not be optimal for the management of the multifactorial complexity of CFRD. This includes minimal insulin requirements, high risk of hypoglycemia, exacerbations and concomitant steroid therapies, gastroparesis and intestinal and nutritional recommendations.

Sensor augmented pump (SAP) therapy is increasingly accepted as gold standard therapy for people with type 1 diabetes (20–24) and continuous glucose monitoring (CGM) has been validated in people with CFRD (25). CGM-derived parameters of glucose variability have been associated with CF outcomes such as respiratory function and body mass index (BMI) (14, 26–29), but data supporting the benefits of diabetes technology in this populations are still scarce and based on small samples (30–32). Despite its potential advantages, SAP therapy was shown to be less frequently used (4.1% vs 17.7%) and more frequently discontinued (30.0% vs 12.7%) in people with CFRD as compared with those with type 1 diabetes (33).

This study was designed to evaluate the potential impact of insulin therapy optimization with SAP over MDI therapy on glycemic control and body composition in individuals with CFRD requiring insulin treatment.

## 2 Research design and methods

### 2.1 Design

We conducted a non-randomized clinical trial including adult (>18 years) individuals with CFRD on insulin treatment and with HbA1c ≥ 5% (31 mmol/mol). Exclusion criteria were: pregnancy, advanced renal disease, defined by estimated glomerular filtration rate < 30 mL/min/1.73 m^2^, and heart failure.

From November 2020 to November 2021, 82 people with CF attending the Diabetes Unit of the Fondazione IRCCS Ca’ Granda Ospedale Maggiore Policlinico in Milano were evaluated for eligibility.

At the screening visit, all subjects were on MDI insulin therapy and, after 2-week run-in period, they underwent an enrollment visit which included clinical, anthropometric and biochemical assessments. Glucose profiles were evaluated with data downloaded from flash glucose monitoring (FGM)/CGM devices. All participants were proposed to participate in a structured preparatory educational program (training to SAP therapy and carbohydrates counting), structured in 4 face-to-face meetings and performed by a multidisciplinary staff (i.e. nutritionist, diabetologist and nurse). After completing the training, all participants were offered to switch from MDI to SAP therapy. Participants who agreed switched to SAP therapy and those who refused continued MDI-scheme insulin therapy. Subjects from both groups had outpatient visits every 3 months according to standard of care. Protocol visits were performed 6, 12 and 24 months after enrollment.

The study complies with the Declaration of Helsinki. The research protocol was approved by the Ethics Committee of the Fondazione Istituto di Ricovero e Cura a Carattere Scientifico (IRCCS) Ca’ Granda Ospedale Maggiore Policlinico (study number 4166, ID 89006) and has been registered on ClinicalTrials.gov (ClinicalTrials.gov Identifier: NCT04379726). A written informed consent was provided by each participant.

**Figure 1:**
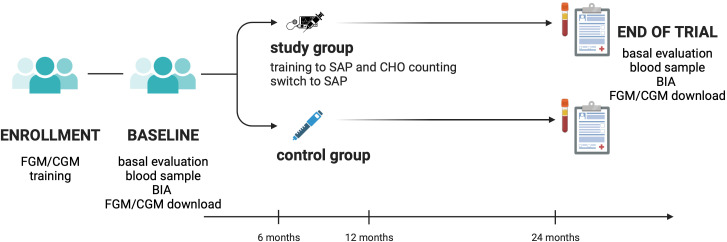
Study flow chart. CGM, continuous glucose monitoring; CHO, carbohydrates; FGM, flash glucose monitoring; BIA, bio-impedance analysis; SAP, sensor-augmented pump; CHO, carbohydrates.

Study flow chart is reported in **Figure 1**.

### 2.2 Measurements

At screening, participants already wore and actively used a FGM/CGM system (Abbott Freestyle Libre 1, Chicago, IL, USA; Abbott Freestyle Libre 2, Chicago, IL, USA; Dexcom G4, San Diego, CA, USA; Dexcom G5, San Diego, CA, USA; Dexcom G6, San Diego, CA, USA; Medtronic Guardian 3, Minneapolis, MN, USA) according to standard of care, at least 3 months prior to baseline. All participants were trained on the use of FGM/CGM system by our medical staff in order to provide a complete 14-day glucose monitoring at each study visit, when they underwent anthropometric evaluation and blood testing including fasting glycaemia (mg/dL, hexokinase) and HbA1c (% and mmol/mol; HPLC). At enrollment and end-of-study visit, body composition was assessed through a tetrapolar single frequency (50 kHz) bioelectrical impedance analyzer (Akern BIA 101 BIVA, Pontassieve, FI, Italy). Data obtained from bioimpedance were analyzed with the software BodyGram Plus. With this method we could estimate fat-free mass (FFM) and fat mass (FM).

Baseline demographic and clinical characteristics, including sex, age, organ transplantation receipt, percent predicted forced expiratory volume in one second (ppFEV1), assessed at the last pulmonary function tests, and daily insulin requirements (IU/kg of body weight/day) were recorded.

Data from FGM/CGM were downloaded using the dedicated software (Medtronic Carelink, Minneapolis, MN, USA; Abbott Libreview, Chicago, IL, USA, Dexcom Clarity, San Diego, CA, USA). Glucose control was evaluated using the following metrics: mean glucose levels (mg/dL), time in range (TIR, %), assuming a 70-180 mg/dlL range according to the American Diabetes Association Standards of Medical Care 2022 (34), time above range (TAR, %) and time below range (TBR, %).

Insulin pumps (Medtronic MiniMed 640G, Minneapolis, MN, USA; Medtronic MiniMed 670G, Minneapolis, MN, USA; Tandem T:slim X2, San Diego, CA, USA; Roche Accu Chek Insight, Basel, CH; Roche Accu Chek Solo, Basel, CH; Insulet Omnipod, Acton, MA, USA; Ypsomed Ypsopump, Burgdorf, CH) and glucose monitors were chosen among the devices currently available in Italy for SAP therapy according to standard of care and individuals everyday life needs.

### 2.3 Study outcomes

The primary endpoint was the change from baseline in HbA1c, whereas secondary endpoints were changes in mean glucose from CGM, TIR, TAR, TBR, weight, BMI, fat-free mass (FFM), fat mass (FM) (in kg or as percent of body weight) and ppFEV1.

### 2.4 Statistical analysis

Overlap weighting was used to address the imbalance in baseline characteristics between treatment groups (35). This technique uses the propensity score to assign weights to each patient that are proportional to the probability of the patient being assigned to the opposite treatment group. The propensity score was estimated through a logistic regression model including the following predictors: sex, age, lung transplantation, and baseline ppFEV1, BMI, FM (% of body weight), insulin requirement, HbA1c, mean glucose and TBR. Overlap weighting has several advantages compared to other weighting options, such as inclusion of all available participants, handling of extreme propensity scores, and exact balance for the mean of all covariates included in the propensity score model. Balance was evaluated using the standardized mean differences (SMD) and values <0.1 were considered indicative of good balance.

Changes in study outcomes from baseline values, between-group differences and corresponding 95% confidence intervals (CI) were estimated using weighted mixed-effects regression models. The weights were obtained from the procedure described above. These models were also used to plot the estimated means at different time points for both groups. A linear mixed-effects quantile regression model was used to estimate the conditional median of the TBR due to the high degree of asymmetry in the distribution of this outcome (36). Models were also adjusted for baseline variables which showed a residual imbalance after weighting. The corresponding 95% confidence intervals (CI) were obtained by bootstrap resampling (n=1000), using the percentile method.

The statistical significance of the between-group differences was evaluated using the likelihood ratio test, testing the significance of the treatment by time interaction.

To adjust for multiple testing, the Benjamini and Hochberg’s False Discovery Rate procedure was used (37). All statistical tests were two-sided with α=0.05.

The analysis was performed using R version 4.3.0 (2023-04-21 ucrt). The “lmer” function from the “lme4” package and the “lqmm” function from the “lqmm” package were used to fit the linear mixed-effects regression models and the quantile mixed-effects regression models, respectively. **Figure 1** was created with Biorender.com (38).

## 3 Results

Forty-six participants (mean age: 35.6 years, range: 21-56) agreed to participate in the educational program and at the end of the program 26 of them refused to switch to SAP therapy and continued with MDI therapy, while 20 switched to SAP.

No participants in either group experienced severe hypoglycemia or diabetic ketoacidosis.

People who switched to SAP used the following SAP devices: 10 Medtronic 670/780, 6 Omnipod, 3 Tandem t:slim, and 1 Accu-Chek Solo. Additionally, 13 devices had a closed loop system, and 7 had a patch pump. None of the participants were on enteral nutrition or were taking energy supplements.

**Table 1** provides a comparison of their baseline demographic and clinical characteristics before and after weighting. Before weighting, participants in the SAP group were more likely females had comparable ages and lung function, while they had a higher BMI and body fatness, lower fasting glucose and insulin requirement as compared to people in the MDI group. Seven participants (35.0%) in the SAP group and 17 participants (65.4%) in the MDI group had received bilateral lung transplantation before enrollment and their steroid therapy dosage remained unchanged for 4 weeks prior to screening visit. Five participants in the SAP group and none in the MDI group were receiving the CFTR modulator therapy lumacaftor/ivacaftor at enrollment. Glycemic control was similar between groups, as indicated by the comparable mean values of HbA1c. After weighting, we obtained two perfectly balanced groups for the measured baseline characteristics, except for a residual imbalance in the frequency of CFTR modulator therapy.

**Table 1:** Baseline characteristics between treatment groups before and after weighting.

Characteristic	Before overlap weighting^a^			After overlap weighting^b^		
	MDI (N=26)	SAP (N=20)	SMD	MDI (N=26)	SAP (N=20)	SMD
Male sex	16 (61.5)	7 (35.0)	-0.54	46.0	46.0	0
Age (years)	35.7 (8.6)	35.5 (8.8)	-0.02	35.3	35.3	0
ppFEV1	70.0 (28.8)	69.9 (27.3)	0	69.2	69.2	0
Lung transplantation	17 (65.4)	7 (35.0)	-0.62	50.1	50.1	0
Height (cm)	165.1 (8.6)	163.5 (9.0)	-0.18	163.5	164.3	0.08
Weight (kg)	61.1 (10.7)	61.8 (13.7)	0.05	60.8	61.7	0.07
BMI (Kg/m^2^)	22.3 (2.6)	22.9 (3.7)	0.20	22.6	22.6	0
FFM (Kg)	51.2 (9.7)	48.2 (10.3)	-0.29	49.5	49.7	0.02
FM (Kg)	10.0 (6.8)	13.5 (6.9)	0.52	11.3	12.0	0.09
FM (% of body weight)	16.0 (9.4)	21.2 (8.9)	0.57	18.3	18.3	0
CFTR modulator therapy^c^	0	5 (25.0)	0.80	0	10.0	0.42
Daily insulin requirement (IU/Kg)	0.75 (0.44)	0.40 (0.22)	-1.02	0.48	0.48	0
Fasting glucose (mg/dL)	186 (103)	152 (71)	-0.38	157	154	-0.04
Mean glucose (mg/dL)	174 (43.5)	167 (44)	-0.15	169	169	0
HbA1c (%)	7.7 (1.8)	7.8 (1.8)	0.05	7.7	7.7	0
TIR (%)	58.6 (19.5)	61.3 (18.5)	0.14	62.3	60.9	-0.08
TAR (%)	36.7 (19.6)	32.5 (18.4)	-0.22	33.1	31.8	-0.08
TBR (%)	5.4 (3.9)	5.2 (3.2)	-0.08	5.6	5.6	0

BMI, Body mass index; FM, Fat mass; FFM, Fat-free mass; IU, International units; MDI, Multiple daily injection; ppFEV1, percent of predicted forced expiratory volume in one second; SAP, Sensor augmented pump; SMD, Standardized mean difference; TAR, Time above range; TBR, Time below range; TIR, Time in range.

^a^ Data for MDI and SAP groups are presented as means (standard deviations) for continuous variable and counts (percentages) for categorical variables.

^b^ Data for MDI and SAP groups are presented as means for continuous variables and percentages for categorical variables. After overlap weighting, a single individual no longer represents a single data entity and thus raw counts are not reported.

^c^ Lumacaftor/Ivacaftor.

All participants completed the last study visit, though HbA1c was not measured in one patient in the MDI group.

**Figure 2:**
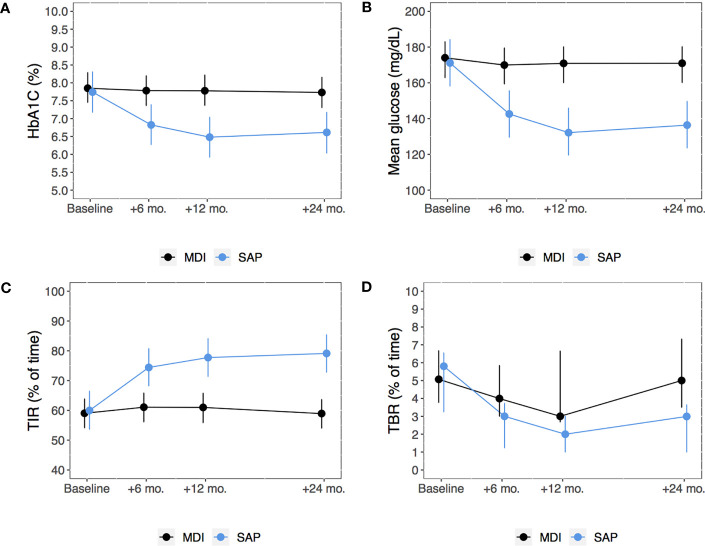
Model-based means of HbA1c **(A)**, mean glucose from continuous glucose monitoring **(B)** and TIR **(C)** and model-based median TBR **(D)** from baseline through 24 months among subjects in the MDI versus SAP therapy group. Points estimates (dots) and 95% confidence intervals (bars) were estimated using linear mixed-effects regression models or mixed-effects quantile regression model (for TBR). MDI, Multiple daily injections; SAP, Sensor augmented pump; TBR, Time below range; TIR, Time in range.

In the SAP group, HbA1c decreased after 6 months from baseline and this reduction persisted over the study period, while it remained substantially unchanged in the MDI group (**Figure 2A**). **Table 2** gives the estimated changes from baseline in HbA1c in both groups. Between-group difference in HbA1c at the end of the study (24 months from enrollment) was -1.0% (-11 mmol/mol) (95% CI: -1.5; -0.5).

**Table 2:** Changes in study outcomes over 24 months among patients in the MDI group and those undergoing SAP therapy, along with the corresponding between-group differences.

Study outcome	Time (months)	Change in the MDI group (95% CI) ^a^	Change in the SAP group (95% CI) ^a^	Between-group difference (95% CI) ^a^	*p*-value ^b^
HbA1c (%)	6	-0.1 (-0.4; 0.3)	-0.9 (-1.3; -0.5)	-0.9 (-1.4; -0.3)	<0.001
	12	-0.1 (-0.4; 0.3)	-1.3 (-1.6; -0.9)	-1.2 (-1.7; -0.7)	
	24	-0.1 (-0.5; 0.3)	-1.1 (-1.5; -0.8)	-1.0 (-1.5; -0.5)	
Mean glucose (mg/dL)	6	-4.0 (-13.5; 5.4)	-28.6 (-37.2; -19.9)	-24.5 (-37.3; -11.7)	<0.001
	12	-3.1 (-12.8; 6.6)	-39.0 (-48.2; -29.8)	-35.9 (-49.2; -22.6)	
	24	-3.1 (-11.7; 5.6)	-34.8 (-43.4; -26.2)	-31.7 (-44.0; -19.5)	
TIR (% of time)	6	2.0 (-1.9; 6.0)	14.4 (10.6; 18.2)	12.4 (7.0; 17.9)	<0.001
	12	2.0 (-2.1; 6.0)	17.8 (14.0; 21.6)	15.8 (10.3; 21.4)	
	24	-0.1 (-3.9; 3.7)	19.2 (15.4; 23.0)	19.3 (13.9; 24.7)	
TAR (% of time)	6	-2.2 (-6.2; 1.9)	-8.8 (-12.7; -4.9)	-6.6 (-12.3; -1.0)	<0.001
	12	-1.2 (-5.4; 3.0)	-11.6 (-15.5; -7.7)	-10.4 (-16.2; -4.7)	
	24	-1.3 (-5.3; 2.6)	-15.1 (-19.1; -11.2)	-13.8 (-19.3; -8.2)	
TBR (% of time)	6	-0.1 (-0.4; 0.3)	-0.9 (-1.3; -0.5)	-0.9 (-1.4; -0.3)	0.886
	12	-0.1 (-0.5; 0.3)	-1.3 (-1.6; -0.9)	-1.2 (-1.7; -0.7)	
	24	-0.1 (-0.5; 0.3)	-1.1 (-1.5; -0.7)	-1.0 (-1.6; -0.5)	
Weight (Kg)	6	-0.7 (-1.9; 0.5)	0.7 (-0.5; 1.9)	1.4 (-0.3; 3.1)	0.037
	12	-0.9 (-2.1; 0.3)	1.3 (0; 2.5)	2.2 (0.4; 3.9)	
	24	-1.0 (-2.3; 0.2)	1.4 (0.2; 2.6)	2.4 (0.7; 4.2)	
BMI (kg/m^2^)	6	-0.3 (-0.8; 0.2)	0.3 (-0.2; 0.7)	0.6 (-0.1; 1.2)	0.031
	12	-0.4 (-0.8; 0.1)	0.5 (0; 1.0)	0.9 (0.2; 1.5)	
	24	-0.5 (-0.9; 0.0)	0.5 (0.1; 1.0)	1.0 (0.3; 1.7)	
FFM (Kg)	24	-0.3 (-1.9; 1.4)	5.2 (3.6; 6.9)	5.5 (3.2; 7.8)	<0.001
FM (Kg)	24	-0.5 (-2.2; 1.2)	-3.9 (-5.6; -2.3)	-3.4 (-5.8; -1.0)	0.011
FM (% of body weight)	24	-0.7 (-3.0; 1.7)	-6.2 (-8.5; -3.8)	-5.5 (-8.8; -2.2)	0.004
ppFEV1	24	-2.3 (-9.4; 4.8)	6.6 (-0.3; 13.4)	8.8 (-1.0; 18.7)	0.093

BMI, Body mass index; CI, Confidence interval; FFM, Fat-free mass; FM, Fat mass; ppFEV1, percent of predicted forced expiratory volume in one second; TAR, Time above range; TBR, Time below range; TIR, Time in range.

^a^ Data are estimated means (95% CI) obtained through weighted linear mixed-effects regression models. When the response variable was TBR data are presented as medians (95% CI) and were obtained from a quantile mixed-effects regression model. All models included terms for treatment group, time and group by time interaction and were adjusted for use of CFTR modulator at enrollment.

^b^ p-values for secondary outcomes were adjusted for multiple testing.

In people who switched to SAP, mean glucose from CGM significantly decreased and TIR increased, while no significant changes were observed in participants in the MDI group (**Figures 2B, C**). TBR did not significantly change during the study period in both groups (**Figure 2D**).

**Figure 3:**
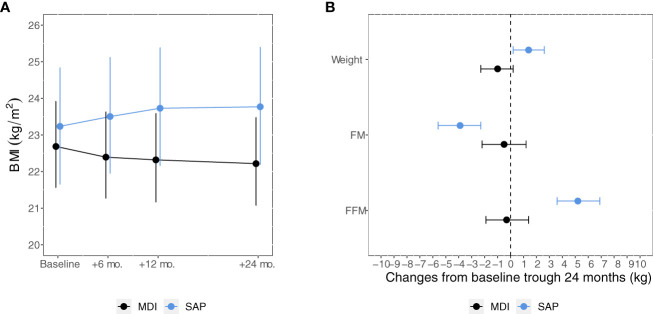
Model-based means of BMI **(A)** from baseline through 24 months and 24-month changes in weight, FM and FFM **(B)** among participants in the MDI versus SAP therapy group. Points estimates (dots) and 95% confidence intervals (bars) were estimated using linear mixed-effects regression models. FM, Fat mass; FFM, Fat-free mass; MDI, Multiple daily injections. SAP, Sensor augmented pump.

People who switched to SAP also had a significant increase in body weight, BMI and fat-free mass and a reduction in fat mass (**Figures 3A, B**). Changes in ppFEV1 were not significantly different between groups. Estimates of the absolute changes from baseline at each study visit and between-group differences are reported in **Table 2**.

## 4 Discussion

In this non-randomized controlled clinical trial, the use of SAP therapy in insulin-requiring adults with CFRD was associated with a significant improvement in glycemic control, expressed as HbA1c, percent of TIR, and mean glucose levels. In addition, SAP therapy induced significant and beneficial changes in body composition with relevant increases in fat-free mass.

Scully KJ et al. recently demonstrated a significant improvement in mostly all CGM-derived glucose control parameters in a 3-month follow-up period after switching 13 adolescents and adults with CFRD from MDI to insulin pumps (32). In that study, average glucose decreased from 189 to 159 mg/dL and TIR increased from 54% to 70%. In our larger trial, we were also able to demonstrate a clinically meaningful difference in HbA1c of ∼1% (12.1 mmol/mol) in the group who switched to SAP therapy as compared to people who continued MDI therapy, with a mean HbA1c value in the SAP group that was below the ADA recommended target of <7% (53 mmol/mol). The reduction in HbA1c was mirrored by an improvement in CGM-derived glucose control parameters with mean glucose falling below 140 mg/dL and TIR exceeding the threshold of 70% after 6 months in SAP treatment. These improvements in glycemic control extended to 2 years, thus indicating a persistent superiority of SAP over MDI.

We also demonstrated that an optimized glycemic control, obtained with such a tailored therapy, can be an effective strategy in obtaining a beneficial impact on body composition, as shown by the increased fat-free mass and reduced fat mass in participants who switched to SAP. This effect along with the improved glycemic control is of major relevance in people with CF, and especially in those with CFRD in whom the catabolic state induced by insulin insufficiency increase the risk of poor nutritional status (7). In CF individuals, fat-free mass is an accurate indicator of nutritional status and its depletion is associated with poor lung function and disease-related morbidity and mortality (39, 40).

To our knowledge, this is the first study that provides evidence of the beneficial effects of SAP in terms of both glycemic control and body composition in people with CFRD. The main limitation is the non-randomized study design leading to important differences in the baseline characteristics of the two groups, which, however were addressed by using overlap weighting, an efficient procedure that mimics the attributes of randomized clinical trials. Furthermore, impaired glycemic control is associated with the progression of lung disease and impaired nutritional status (41). Conversely, frequent respiratory infections and exacerbations can also negatively affect insulin sensitivity and body composition (42). However, our study did not collect this information and was not adequately powered to explore the potential role of changes in pulmonary infections as a mechanism for improved glycemic control and body composition. Finally, due to the limited number of subjects enrolled, we were unable to test whether the different SAP devices used by the study participants yielded different results.

In conclusion, this study shows that SAP therapy leads to a significantly better glycemic control than MDI therapy, resulting in increased fat-free mass and suggesting that it may be particularly useful in treating people with CFRD. However, further studies are needed to assess the effect of optimized glucose control with SAP therapy and the associated improvement in nutritional status on long-term outcomes, such as lung function, CF- and CFRD related morbidity, quality of life, and mortality in these individuals.

## Data availability statement

The data presented in this article are not publicly available due to legal and privacy restrictions, specifically concerning patient confidentiality and participant privacy. Requests to access data should be directed to the corresponding author.

## Ethics statement

The studies involving humans were approved by the Ethics Committee of the Fondazione IRCCS Ca’ Granda Ospedale Maggiore Policlinico Milano. The studies were conducted in accordance with the local legislation and institutional requirements. Written informed consent for participation in this study was provided by the participants’ legal guardians/next of kin.

## Author contributions

VG conceptualized and designed the study, drafted the manuscript and collected data. GA carried out the statistical analyses and critically reviewed the manuscript. LP, LZ, AGr, LCM, VRo, AGa, and VRe collected data. EO, VD, and FB coordinated and supervised data collection, and critically reviewed the manuscript. All authors approved the final manuscript as submitted and agree to be accountable for all aspects of the work. VG is the guarantor of this work and, as such, had full access to all the data in the study and takes responsibility for the integrity of the data and the accuracy of the data analysis.
